# A Narrow Straight Microchannel Array for Analysis of Transiting Speed of Floating Cancer Cells

**DOI:** 10.3390/mi13020183

**Published:** 2022-01-26

**Authors:** Jifeng Ren, Yi Liu, Wei Huang, Raymond H. W. Lam

**Affiliations:** 1 School of Biomedical Engineering, Capital Medical University, Beijing 100069, China; 2 Beijing Key Laboratory of Fundamental Research on Biomechanics in Clinical Application, Capital Medical University, Beijing 100069, China; 3 Department of Biomedical Engineering, City University of Hong Kong, Kowloon, Hong Kong 999077, China; yiliu94-c@my.cityu.edu.hk (Y.L.); whuang42-c@my.cityu.edu.hk (W.H.); 4 City University of Hong Kong Shenzhen Research Institute, Shenzhen 518057, China; 5 Centre for Biosystems, Neuroscience, and Nanotechnology, City University of Hong Kong, Kowloon, Hong Kong 999077, China

**Keywords:** cell transiting speed, theoretical analysis, computational fluid dynamics, microfluidic chip, narrow microchannel

## Abstract

Investigating floating cells along a narrow microchannel (e.g., a blood vessel) for their transiting speeds and the corresponding roles of cell physical properties can deepen our understanding of circulating tumor cells (CTCs) metastasis via blood vessels. Many existing studies focus on the cell transiting process in blood vessel-like microchannels; further analytical studies are desired to summarize behaviors of the floating cell movement under different conditions. In this work, we perform a theoretical analysis to establish a relation between the transiting speed and key cell physical properties. We also conduct computational fluid dynamics simulation and microfluidic experiments to verify the theoretical model. This work reveals key cell physical properties and the channel configurations determining the transiting speed. The reported model can be applied to other works with various dimensions of microchannels as a more general way to evaluate the cancer cell metastasis ability with microfluidics.

## 1. Introduction

Cancer metastasis involves cancer cells establishing new colonies from a primary tumor; this process can lead to poor prognostic and high mortality risks [1,2,3,4]. Tumor cells extravasating successfully become floating tumor cells in blood vessels, which is a decisive step in the formation of a secondary tumor [5]. Such circulating tumor cells (CTCs) have been considered as an indicator in cancer diagnosis such as prognostic markers [6,7,8]. It is well known that the metastasis ability of CTCs is associated with physical properties at the single-cell level such as deformability [9] and moving/transiting speed in constricted regions [10]. Among these physical properties, the transiting speed in blood vessels [11], as well as the flow characteristics of the surrounding liquid [12], play important roles in determining the cancer metastasis ability. CTCs can undergo large deformations in constricted narrow regions, implicating the key role of cell deformability. The cell deformability influences not only the initial process entering the bloodstream [13,14] but also the transiting process of CTCs [15]. Therefore, studying the movement of floating cancer cells along a constricted microchannel can provide insights for studying cancer metastasis.

Microfluidics has been recognized as an effective approach for studying the floating cell in microchannels, overcoming the technical challenges for observing cells in vivo, e.g., low throughput [16] and low imaging quality [11]. Microfluidic methods are able to study the physical properties of individual floating cells in blood vessels [17,18]. Noticing floating cells transiting speed is an important physical property for CTC metastasis, the transiting speed for different internal environmental structures can also be studied with microfluidics. For instance, some researchers investigated the CTC transiting speed in a three-dimensional (3D) microchannel network with various geometries [19]. Recent work reported that CTC clusters are able to enter narrow microchannels in the chain-like organizations, and the transiting speed in capillaries relates to the metastatic potential [10].

In addition, many studies are interested in applying analytical methods to investigate the cancer cell movement along constricted regions. For example, some researchers applied two-dimensional (2D) numerical modeling to analyze the process of cancer cells entering along microchannels based on the common dimensionless parameters [14]. Whereas the duration for a cell entering a narrow channel is largely influenced by the initial conditions including the initial cell speed, the transiting speed after a cell fully entering a microchannel is a more promising indicator. There have been great demands on the prediction of cancer, the cell transiting speed under different flow conditions and channel geometry [20], with clinical relevance to diseases such as thrombus [21] and atherosclerosis [22], for which the corresponding vessels may change their shapes. 

In this work, we perform theoretical analysis, simulation, and experiments of cell movements along a narrow microchannel. Theoretical modeling and finite element method (FEM) simulation are applied to investigate the transiting speed of floating cells and its relationship with the cell physical properties and microchannel configuration. A 3D two-phase laminar flow is considered in the simulation. We further quantify the cell transiting speed varying with the cell size. We conduct microfluidic experiments to verify the theoretical analysis of different types of breast cells (i.e., MCF-7, MCF-10A, MDA-MB-231) flowing along the microchannels.

## 2. Materials and Methods

### 2.1. Device Fabrication

The microfluidic device contains an array of straight channels, which was fabricated based on soft photolithography. Briefly, a silicon wafer was firstly spin-coated with a negative photoresist (SU-8 2025, Microchem, MA, USA) to generate a patterned layer 30 µm thick as the mold master. After exposure and development, the mold master was then silanized with vaporized (tridecafluoro-1,1,2,2-tetrahydrooctyl)-1-trichlorosilane (Sigma-Aldrich, St. Louis, MO, USA) in a vacuum desiccator > 12 h for more convenient release of PDMS from the mold. To fabricate the PDMS microchannel array, polydimethylsiloxane (PDMS, Sylgard-184, Dow Corning, Midland, MI, USA) prepolymer was prepared by mixing the monomer with the curing agent at a 10:1 volumetric ratio. After degassing, the prepared PDMS pre-polymer was then poured on the silicon mold and thermally cured in an oven at 85 °C for 4 h. Afterward, the fully cured PDMS was stripped off from the silicon mold, punched with holes for inlets and outlets, and bonded onto a glass slide (Citoglas, Nantong, China) by oxygen plasma (PDC-001 HP, Harrick Plasma, Ithaca, NY, USA). 

### 2.2. Simulation

A transiting floating cell in a narrow straight microchannel was considered as a droplet with certain surface tension. The surface tension is determined based on the cell elasticity as described in “Simulation for the Cell Transiting Speed” in Results below. An inlet gage pressure of 16 Pa is set at the channel entrance, whereas the channel exit has a pressure of 0 Pa. Simulation of a two-phase laminar flow was implemented with COMSOL 5.2a (Burlington, MA, USA). No-slip boundary conditions were set to the channel wall surfaces. To reveal the dynamic cell deformation and variations of the related parameters, we adopted a Laplace moving mesh over different time steps. The transiting speeds for different cell types and original diameters ranging from 12 µm to 22 µm were computed. 

### 2.3. Cell Culture

Human breast epithelial (MCF-10A) and human breast cancer (MDA-MB-231 and MCF-7) cell lines were obtained from ATCC (Manassas, VA, USA). MCF-10A cells were maintained in Mammary Epithelial Growth Medium Bulletkit (CC-3150, Lonza, New York, NY, USA), containing a mammary epithelial cell basal medium (MEBM), 0.4% (*v*/*v*) bovine pituitary extract (BPE), 0.1% (*v*/*v*) human epithelial growth factor (hEGF), 0.1% (*v*/*v*) insulin, 0.1% (*v*/*v*) hydrocortisone and 0.1% (*v*/*v*) of a reagent mixed with 30 mg/mL gentamicin and 15 mg/mL amphotericin (GA-1000, Lonza). MDA-MB-231 and MCF-7 cells were cultured in a high-glucose Dulbecco’s modified Eagle’s medium (DMEM; Invitrogen, Carlsbad, CA, USA) supplemented with 10% fetal bovine serum, 0.5 μg/mL fungizone (Invitrogen), 5 μg/mL gentamicin (Invitrogen), 100 units/mL penicillin, and 100 μg/mL streptomycin. All cell types were cultured in an incubator at 37 °C, saturated humidity and 5% CO_2_ in air. When cells reached 80% confluency, fresh 0.25% trypsin-EDTA in PBS was used to resuspend cells before subculture at a cell density of 3 × 10^3^ cells/cm^2^.

### 2.4. Image Capture

Microscopic images were captured by a phase-contrast inverted microscope (TE300, Nikon, Tokyo, Japan) with an sCMOS microscope camera (Zyla 4.2, Andor, Belfast, UK).

### 2.5. Data Processing

Open-source image processing software ImageJ (NIH) was used to analyze the microscopic images, e.g., to measure diameter *D* of undeformed cells.

## 3. Results

### 3.1. Device Design

**Figure 1:**
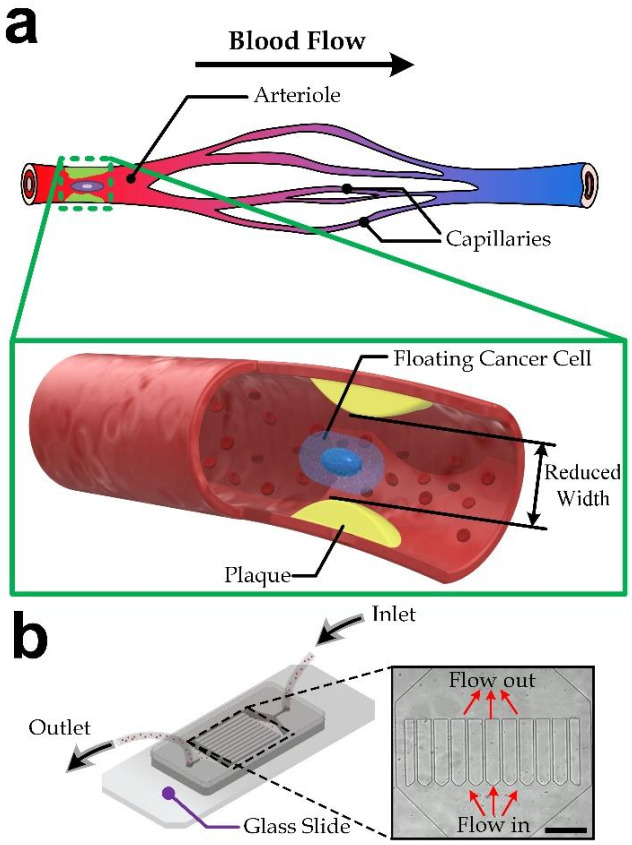
(**a**) Illustration of a circulating tumor cell (CTC) flowing in a human blood vessel. Inset: an enlarged diagram at the atherosclerosis site. (**b**) The microfluidic microchannel array device. Inset: microscopic image of microfluidic channels, scale bar: 200 µm.

We designed and fabricated an array of straight micro-channels to investigate movements of floating cells with large deformation along constricted regions, analogous to the small vessels such as arteriole, as described in Figure 1a. Such movement of deformed cells is similar to the case of CTCs flowing along blood vessels after intravasation [23]. The device layout is shown in Figure 1b. Each microchannel has a cross-section geometry of 10 µm × 30 µm (width × height). The channel length is 380 µm. There are ten microchannels in parallel, with two bypass side-microchannels with a different width of 40 µm, to maintain the pressure difference across the microchannel structure to be more consistent. Standard soft lithography process (see Methods) is applied for the device fabrication.

### 3.2. Modeling of a Deformed Cell Transiting along a Straight Channel

**Figure 2:**
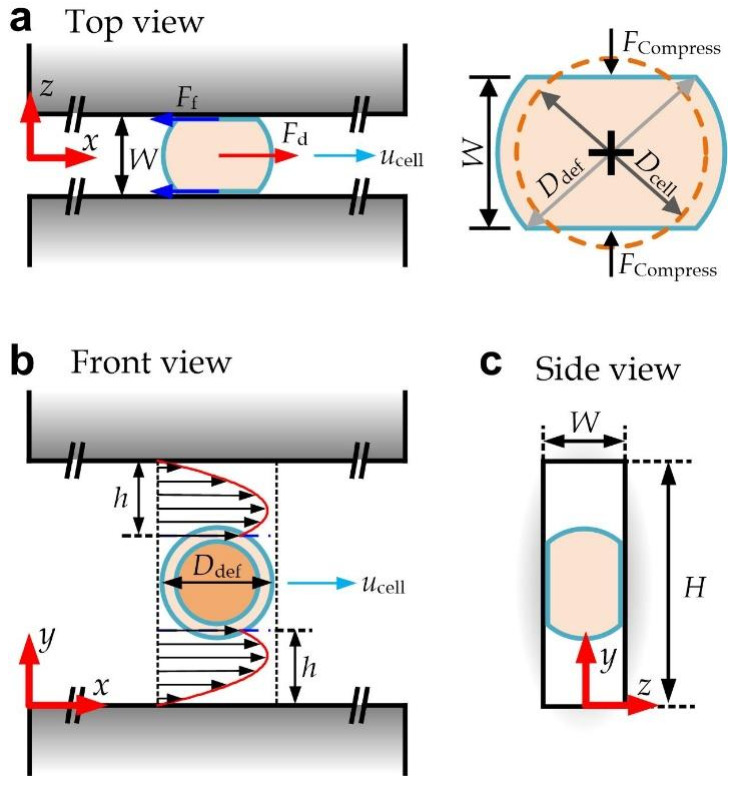
Dimensions and three-view diagrams of a deformed cell flowing in a straight microchannel: (**a**) top view; (**b**) front view; (**c**) side view.

We develop a theoretical model for the cell transiting speed along a constricted microchannel (width *W* along the z-direction, and height *H* along the y-direction) and its relationship with key cell physical properties and microchannel configurations, as illustrated in Figure 2. When a cell with a diameter of *D*_cell_ enters a narrow microchannel with *W* < *D*_cell_ such that the cell must deform to fit inside the channel. The deformed shape of a cell can be approximated as a sphere with its top and bottom chopped along contacting surfaces on both sides of the channel. As the cell volume should maintain during the deformation, the corresponding diameter of the deformed spheric surface (*D*_def_) can be expressed as [24]:

(1) $$D_{\mathrm{def}}=\sqrt{\frac{2D_{\mathrm{cell}}^{3}}{3W}+\frac{W^{2}}{3}}$$

Meanwhile, the channel height is still larger than the deformed cell diameter, leaving vacant regions on both sides of the cell along the channel height direction. Apparently, liquid can still flow via these vacant regions. Each vacant region is then considered as a rectangular slit with a width *W*, a length *D*_def_, and an equivalent height *h*, which can be expressed as

(2) $$h=\frac{\zeta }{2}\left(H-\frac{D_{\mathrm{def}}}{\kappa }\right)$$

where *κ* is a geometric parameter; and *ζ* is a correction factor. *ζ* will be determined by fitting the experimental data with the model described at the end of this section. To approximate the geometric parameter *κ*, we consider the value of *h* such that the total volume of both slits equals that of the vacant regions:

(3) $$D_{\mathrm{def}}WH-\frac{4}{3}\pi {\left(\frac{D_{\mathrm{cell}}}{2}\right)}^{3}=2D_{\mathrm{def}}Wh$$

Hence,

(4) $$\kappa =\chi \left[\frac{4}{\pi }+\frac{2}{\pi }{\left(\frac{W}{D_{\mathrm{cell}}}\right)}^{3}\right]$$

where *χ* is a correction factor. *χ* will be determined by fitting the experimental data. In each slit, the velocity profile *u* has the simplified governing equation:

(5) $$\eta \frac{d^{2}u}{dy^{2}}=\frac{dp}{dx}$$

where *x* is the direction along the channel length, *y* is the direction along the channel height, *dp*/*dx* is the pressure gradient, and *η* is the liquid viscosity (~10^−3^ Pa·s for water). If we further consider *y* = 0 is at the lower channel wall in the front view as shown in Figure 2. The boundary conditions for the corresponding slit are *u* = 0 at *y* = 0 and *u* = *u*_cell_ at *y* = *h*, where *u*_cell_ is the cell transiting speed. Then, the velocity can be expressed as:

(6) $$u=\frac{1}{2\eta }\cdot \frac{dp}{dx}y^{2}+\left(\frac{u_{\mathrm{cell}}}{h}-\frac{1}{2\eta }\cdot \frac{dp}{dx}h\right)y$$

The effective shear stress over the lower side of the cell surface as indicated in the front view of Figure 2 can then be approximated as:

(7) $$\tau =-\eta {\left.\frac{du}{dy}\right|}_{y=h}=-\frac{1}{2}\frac{dp}{dx}h-\eta \frac{u_{\mathrm{cell}}}{h}$$

The drag force *F*_d_ acting on the deformed cell can then be approximated as

(8) $$F_{d}=\int_{A}\tau dA\approx WD_{\mathrm{def}}\left(\gamma h-2\eta \frac{u_{\mathrm{cell}}}{h}\right)$$

where *γ* = −*dp*/*dx* represents the average pressure drop per unit length along the slits.

We further consider the balance of the drag force *F*_d_ and the friction between the cell and both channel walls *F*_f_, the *F*_f_ can be calculated as

(9) $$F_{f}=2\mu F_{\mathrm{compress}}$$

where *μ* (~0.00025 [25]) is the friction coefficient; *F*_compress_ is the compressive force from the channel sidewalls, acting on the deformed cell. We applied a constant Poisson’ ratio for three breast cell lines since the Poisson’s ratio varies little between breast tumor cells and benign cells [26]. *F*_compress_ can be approximated by the Hertz contact theory [24]:

(10) $$F_{\mathrm{compress}}=\frac{E}{3\left(1-\upsilon^{2}\right)}\sqrt{D_{\mathrm{cell}}{\left(D_{\mathrm{cell}}-W\right)}^{3}}$$

where *ν* (~0.499 [27]) is the Poisson’s ratio of the cell; *E* is the whole-cell elasticity (0.500 kPa for MCF-10A [28], 0.300 kPa for MCF-7 [28], and 0.285 kPa for MDA-MB-231 [29]). Therefore, the cell transiting speed *u*_cell_ becomes:

(11) $$u_{\mathrm{cell}}\;\sim \;\frac{\gamma }{2\eta }h^{2}-\frac{2\mu F_{\mathrm{compress}}}{\eta WD_{\mathrm{def}}}h$$

It should be mentioned that the first and second terms on the right-hand side in the equation above are caused by the viscosity effect and the friction, respectively. In this work, scaling of the viscosity term is much larger than that of the friction term; and hence we can expect that *u*_cell_ is more sensitive to the cell size than cell mechanical properties such as the whole-cell elasticity.

### 3.3. Simulation for the Cell Transiting Speed

We conducted a numerical simulation to compute for the pressure drop along a microchannel as described in Methods. To obtain dynamic cell movement along a microchannel, a moving mesh and auto-remeshing two-phase laminar flow method was applied. The mean dynamic viscosity inside the cell was obtained based on a scaling formula method [30]. The surface tension *T* can be estimated from the compressive force with the relation [31]:

(12) $$T\;\sim \;2A_{\mathrm{cell}}\left(\frac{1}{W}-\frac{1}{D_{\mathrm{def}}}\right)F_{\mathrm{compress}}$$

where *A*_cell_ is the cell surface area, which can be approximated by:

(13) $$A_{\mathrm{cell}}=\frac{\pi }{2}\left(D_{\mathrm{def}}^{2}+2D_{\mathrm{def}}W-W^{2}\right)$$

**Figure 3:**
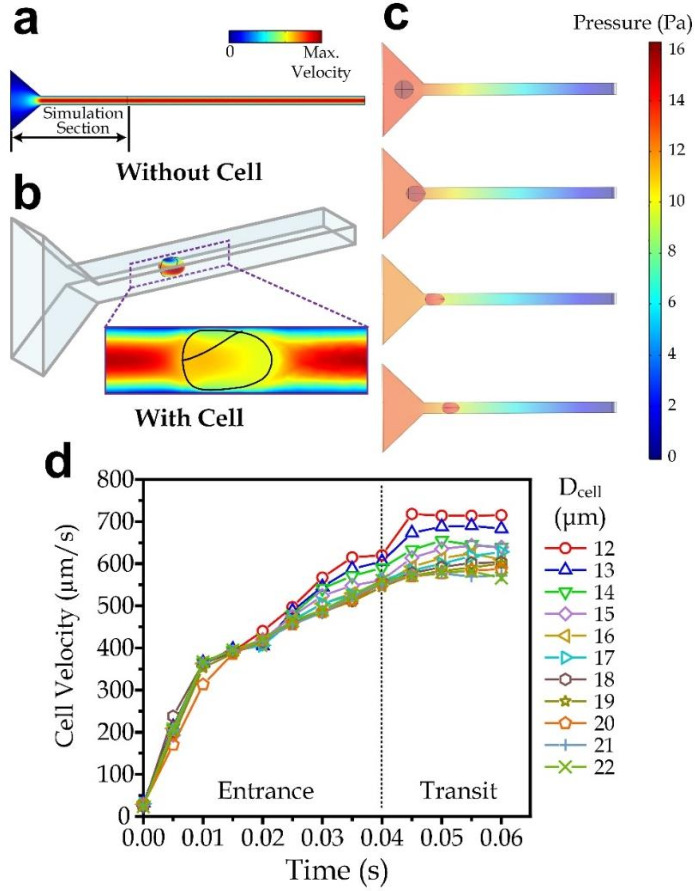
(**a**) Normalized velocity distribution of the whole microchannel without a floating cell. (**b**) Normalized velocity distribution around a cell (undeformed diameter: 16 µm) passing along the center length–width plane. (**c**) Simulated pressure profile. (**d**) Simulated cell velocity against the time since contacting the microchannel, for undeformed cell diameters.

To quantify the fluidic resistance values, laminar flow simulation was adopted in the whole microchannel as shown in Figure 3a. We then obtained the effective fluidic resistance as the ratio of the average pressure values and the flow rates for each flow section. We consider the fluidic resistances for the deformed cell section (*R*_1_) and the remaining channel (*R*_2_).

For demonstration, we analyzed a cell with a 16 µm diameter entering a micro-channel under an inlet gage pressure of 16 Pa. The cell morphology change at different time points is shown in Figure 3b. We performed a simulation to obtain the transiting speeds for cell diameters ranging from 12 μm to 22 μm (Figure 3c). It can be observed that the cell transiting speed along the channel (after 0.05 s) is more stable compared to cell velocity when entering the channel (before 0.04 s). In other words, the transiting speeds for a cell at different positions traveling along the microchannel have a very closer value, implying high repeatability and robustness of the measurement. It can be observed that a larger cell induces a slower cell transiting speed. 

**Figure 4:**
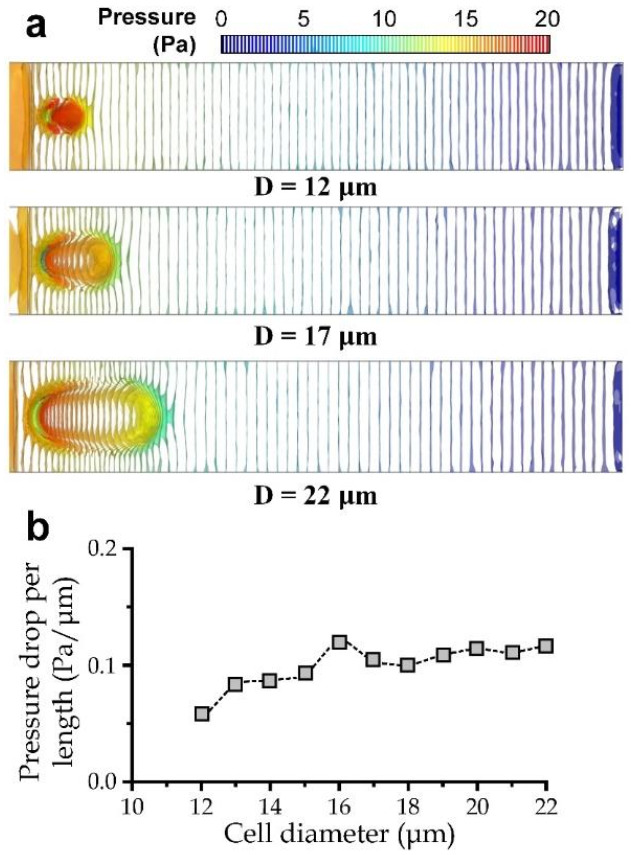
(**a**) Pressure iso-surfaces in the channel front view for a cell with a diameter of 12 μm, 17 μm, and 22 μm. (**b**) Average pressure drop per length along the deformed cell region *γ* for different cell diameters.

Furthermore, we also calculated the average pressure drop per length along the cell region *γ* for different cell diameters. We considered pressure iso-surfaces for the region around the deformed cell in the simulation (Figure 4a) to estimate for *γ*. We summarize the values of *γ* for different cell diameters in Figure 4b, suggesting that *γ* values can be considered as roughly a constant value of ~0.0994 Pa/μm.

### 3.4. Experiment Verification

**Figure 5:**
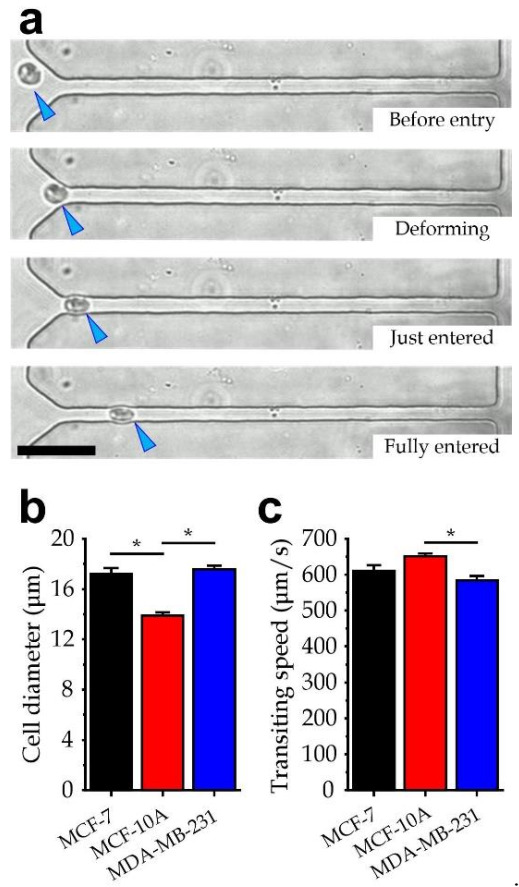
(**a**) Representative images of an MDA-MB-231 cell flowing along a straight microchannel. Scale bar: 50 μm. (**b**) Diameters of the three selected cell types. *N* = 35 for MCF-7, *N* = 25 for MCF-10A, and *N* = 57 for MDA-MB-231. (**c**) Transiting speeds of the three selected cell types. An asterisk represents *p* < 0.05 in Student’s *t*-test.

To further verify the theoretical model, we conducted experiments to inject floating cells along the microfluidic channel array. We adopted three breast cell lines: breast benign cell MCF-10A, breast malignant cancer cell MCF-7, and breast invasive cancer cell MDA-MB-231. We took time-lapsed microscopic images and quantified for the deformed/undeformed diameter and transiting speed for each floating cell passing through any of the narrow microchannels. The cell deformation and transiting process for an MDA-MB-231 transiting along the straight channel is provided in Figure 5a as a demonstration. We summarized the average cell undeformed diameter (Figure 5b) and the cell transiting speed (Figure 5c) for individual cell lines. The results conclude that the transiting speed *u*_cell_ should be negatively correlated with the undeformed cell diameter *D*_cell_, which agrees with both the simulation and the analytical model presented above.

**Figure 6:**
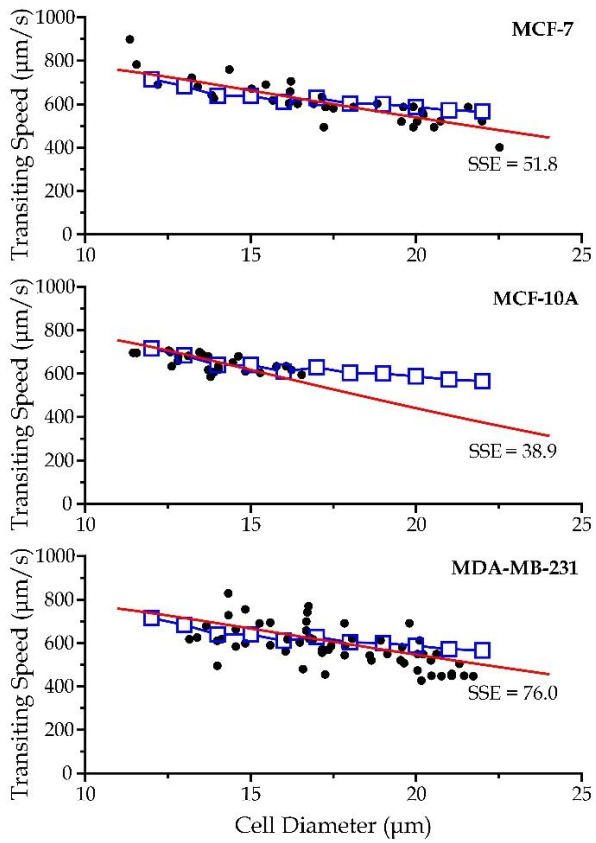
Analytical model predictions (red lines), simulation results (blue boxes), and experimental data (black dots) of the transiting speeds against cell diameters for the three selected cell types. SSE represents for standard error of the estimates.

We then conducted curve-fitting on the analytical model with the experimental results of the transiting speeds using the curve fitting toolbox function in MATLAB R2016b (MathWorks), to determine the scaling factors. The experimental data, simulation results and predicted values by the analytical model for different cell types are presented in Figure 6. The standard errors of the estimates for different cell types have ranges of values much smaller than the range of variation in the transiting speed, indicating that the theoretical prediction can describe the major relationship between the transiting speed and the cell diameter. Overall, the derived analytical model can reflect the trend of cell transiting speed against different cell diameters. Surprisingly, the computed correction factors (*χ*~7.236 and *ζ*~0.2695) are very close for all the three selected cell types, implicating the model can effectively describe the relationship of the transiting speed with the corresponding key cell physical properties, flow conditions and microchannel configurations. 

## 4. Discussion

In fact, the analytical model developed in this work can be further applied to predict the cell transiting speed for other cell types with different ranges of physical parameters (e.g., diameter and elasticity) and microchannel configurations (e.g., width and height); and therefore this model can guide the design of microchannels for a specific cell type flowing within a target range of the transiting speed for any further biosensing applications, including viscoelasticity measurements [32] and characterization of fluorescence signals from the in vivo flow cytometry [33] for CTCs.

In summary, an analytical model describing the cell transiting speed with deformation along a constricted microchannel is successfully established. It suggests that the transiting speed negatively correlates with the cell diameter. We performed a two-phase laminar flow simulation to verify the predicted trend between the transiting speed and the cell diameter. Further, microfluidic experiments using three breast cell lines (MDA-MB-231, MCF-7 and MCF-10A) were also applied to evaluate the simulation results and the analytical model, with good agreements. This study provides the essential relation for determining the transiting speed of a deformed cell passing along a constricted narrow channel. Further applications include cancer prognosis, hemodynamics studies, investigation of vascular disease (e.g., atherosclerosis). Additionally, it can also help determine the microchannel configurations (e.g., width and height) for target cell types with known ranges of physical parameters (e.g., diameter and elasticity) to achieve a transiting speed within a required range for various biosensing applications.
